# Different Dynamic Patterns of β-Lactams, Quinolones, Glycopeptides and Macrolides on Mouse Gut Microbial Diversity

**DOI:** 10.1371/journal.pone.0126712

**Published:** 2015-05-13

**Authors:** Jia Yin, Prabhakar M, Shan Wang, Shuo-Xi Liao, Xin Peng, Yan He, Yi-Ran Chen, Hua-Fang Shen, Jin Su, Ye Chen, Yun-Xia Jiang, Guo-Xia Zhang, Hong-Wei Zhou

**Affiliations:** 1 Department of Neurology, Nanfang Hospital, Southern Medical University, Guangzhou, China 510515; 2 State Key Laboratory of Organ Failure Research, Department of Environmental Health, School of Public Health and Tropical Medicine, Southern Medical University, Guangzhou, China 510515; 3 Department of respiration, Nanfang Hospital, Southern Medical University, Guangzhou, China 510515; 4 Department of Digestive Diseases, Nanfang Hospital, Southern Medical University, Guangzhou, China 510515; Max Rubner-Institut, GERMANY

## Abstract

The adverse impact of antibiotics on the gut microbiota has attracted extensive interest, particularly due to the development of microbiome research techniques in recent years. However, a direct comparison of the dynamic effects of various types of antibiotics using the same animal model has not been available. In the present study, we selected six antibiotics from four categories with the broadest clinical usage, namely, β-lactams (Ceftriaxone Sodium, Cefoperazone/Sulbactam and meropenem), quinolones (ofloxacin), glycopeptides (vancomycin), and macrolides (azithromycin), to treat BALB/c mice. Stool samples were collected during and after the administration of antibiotics, and microbial diversity was analyzed through Illumina sequencing and bioinformatics analyses using QIIME. Both α and β diversity analyses showed that ceftriaxone sodium, cefoperazone/sulbactam, meropenem and vancomycin changed the gut microbiota dramatically by the second day of antibiotic administration whereas the influence of ofloxacin was trivial. Azithromycin clearly changed the gut microbiota but much less than vancomycin and the β-lactams. In general, the community changes induced by the three β-lactam antibiotics showed consistency in inhibiting *Papillibacter*, *Prevotella* and *Alistipes* while inducing massive growth of *Clostridium*. The low diversity and high *Clostridium* level might be an important cause of *Clostridium difficile* infection after usage of β-lactams. Vancomycin was unique in that it inhibited Firmicutes, mainly the genus *Clostridium*. On the other hand, it induced the growth of *Escherichia* and effect lasted for months afterward. Azithromycin and meropenem induced the growth of *Enterococcus*. These findings will be useful for understanding the potential adverse effects of antibiotics on the gut microbiome and ensuring their better usage.

## Introduction

Hippocrates has been quoted as saying “death sits in the bowels” and “bad digestion is the root of all evil” in 400 B.C. [1, 2], showing that the importance of the intestine in human health has been long recognized. Gastrointestinal tract (GIT) is one of the largest interfaces between the outside world and the human internal environment. From mouth to anus, it forms a nine-meter long tube, constituting the body’s second largest surface area, which is estimated to cover approximately 250–400 m^2^ [1]. The microbiota of the GIT represents an ecosystem of the highest complexity. Human gutmicrobiota is believed to be composed of over 50 genera of bacteria [3] accounting for over 500 different species [4]. An adult human GIT is estimated to contain 10^14^ viable microorganisms, which is 10 times the number of eukaryotic cells found within the human body [5]. The GIT microbiota is involved in the stimulation of the immune system, synthesis of vitamins (B group and K), enhancement of GIT motility and function, digestion and nutrient absorption, inhibition of pathogens, metabolism of plant compounds/drugs and production of short-chain fatty acids (SCFAs) and polyamines [6–8].

Bowel toxemia theories eventually evolved into the intestinal dysbiosis hypothesis. The term “dysbiosis” was originally coined by Metchnikoff to describe altered pathogenic bacteria in the gut [8]. Dysbiosis is a state in which the microbial community in the gut produces harm through qualitative and quantitative changes in the intestinal microbiota, bacterial metabolic activity and the local distribution of bacteria [9]. The dysbiosis hypothesis states that the modern diet and lifestyle, as well as the use of antibiotics, have led to the disruption of the normal intestinal microbiota. These factors have led to alterations in bacterial metabolism, as well as the overgrowth of pathogenic microorganisms, which results in the release of potentially toxic compounds that play a role in many chronic and degenerative diseases. An altered intestinal microbiota is now believed to play a role in a myriad of disease conditions, including GIT disorders such as irritable bowel syndrome (IBS) [10] and inflammatory bowel disease (IBD) [11, 12], as well as more systemic conditions such as rheumatoid arthritis (RA) [13] and ankylosing spondylitis [14]. Thus, knowledge of the factors that can cause detrimental changes to the microbiota is becoming increasingly important to the clinician.

Antibiotic use is one of the common and significant cause of major alterations in normal GIT microbiota [1]. It is known that antimicrobial agents not only affect the pathogens to which they are directed but may also impact other members of the intestinal microbiota [15]. The potential for an antimicrobial agent to influence the gut microbiota is related to its spectrum of activity [16], pharmacokinetics, dosage [17], mode of action [18] and length of administration [19].

The major advantages of using next-generation sequencing (NGS) to determine 16S rRNA tags compared to traditional microbial community profiling methods are that it is cost-effective and high throughput [20]. We could obtain the desired microbial community data with NGS, which provides a sufficient tool to study microbial ecology [20].

There is much research regarding the extent of gut microbiome disturbances after antibiotic therapy. The amount of disturbance depends on the spectrum of the agent, the dose, the route of administration, and the pharmacokinetic and pharmacodynamic properties. However, there are very few reports on the effects of different categories of antibiotics on the gut microbiota. In the present work, we studied the extent of the gut microbiome disturbance with different categories of antibiotics in a follow-up study comparing microbial communities before, during and after antibiotic therapy in mice. We also studied the influence of these various categories of anti-microbial agents on the recovery time of the gut microbiome after antibiotic therapy.

## Methodology

### Ethical Statement

All animal studies were conducted after ethical approval from the Institutional Animal Ethics Committee (IAEC), Southern Medical University, Guangzhou.

### Experimental animals

A total of 56 male BALB/c mice were used in this study. They were divided into 6 groups randomly (8 mice per group) with consistent average weights. After pre-feeding for a week, the groups of mice were treated short-term with one of the following for 4 days: Ceftriaxone sodium (CTR), cefoperazone/sulbactam (CPZ), meropenem (MEC), ofloxacin hydrochloride (OFL), vancomycin (VAN), azithromycin (AZI) or a no antibiotic treatment control (CK). The first three antibiotics, CTR, CPZ and MEC, belong to the ß-lactam group, OFL belongs to the quinolones, AZI belongs to the macrolides and VAN belongs to the glycopeptides. For each of the antibiotics, the dosage was calculated based on the human dosage. The dosages (mg/20 gm body weight) given through subcutaneous injection on each day for four days were as follows: CTR- 4.325, CPZ-5.767, MEC-4.325, OFL-1.153, VAN-5.676 and AZI-1.442.

### Sample collection

Fresh fecal samples were collected directly into sterile 2 ml tubes from the anus of healthy adult male BALB/c mice without antibiotic treatment and immediately stored at -80°C until DNA extraction. For experimental animals, stool samples were collected before (I.1, I.2, I.3), during (II.1, II.2, II.3, II.4) and after (III.1, III.2, III.3, III.4, III.5, III.6, III.7, III.14, III.30, III.90) the administration of antibiotics. All samples were immediately stored at -80°C until DNA extraction.

### DNA extraction and PCR amplification

DNA extraction and PCR amplification were performed as described in our previous study [19]. DNA extraction was carried out by a boiling method, which is cost-effective and time saving.

### Processing of sequences and bioinformatics analysis

We used the BIPES pipeline to process raw sequences, and UCHIME [21] was used to screen chimeras. After that, we used a Two-Stage-Clustering (TSC) [22] algorithm to cluster tags into operational taxonomic units (OTUs); the taxonomy assignment of tags and OTUs was performed using the Global Alignment for Sequence Taxonomy (GAST) [23]. The rarefaction curves of observed species, the Shannon index and PCoA analysis were implemented using QIIME based on UniFrac distance [24]. Biomarkers between groups were determined by linear discriminant analysis (LDA) coupled with the effect size measurements (LEfSe) online tool http://huttenhower.sph.harvard.edu/galaxy/. Statistical analysis was performed using SPSS 13.0.

## Results and Discussion

### Antibiotic influence on alpha diversity

**Fig 1:**
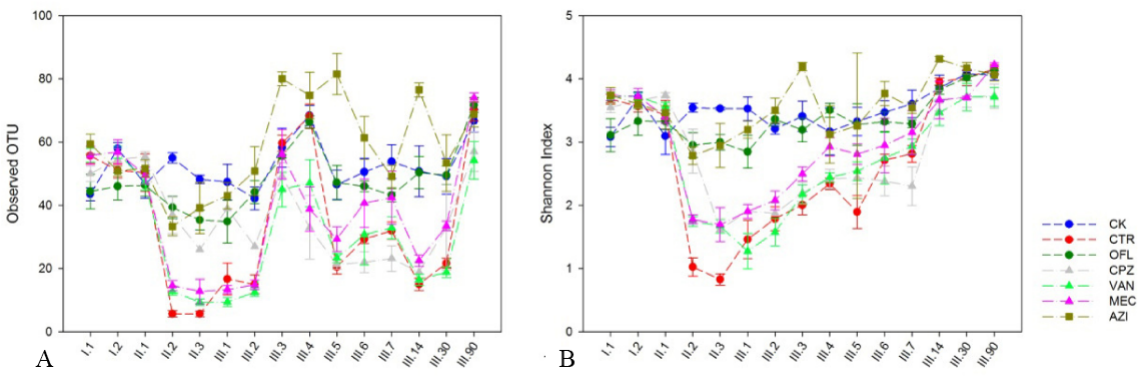
α-diversity indices for different antibiotic groups from the fecal microbiota. A. Chao1 index; B. Shannon index.

Alpha diversity is the diversity of the community within one site (or one sample), that is the number of species and their proportion within one sampling site. We utilized observed OTU and the Shannon index to compare the alpha diversity of different antibiotic groups. Observed OTU is the number of OTUs that are observed in a sample and it is a richness metrics. Shannon index is determined more by the evenness of the microbiota. The observed OTU and Shannon index in the control (CK) group showed no changes during the complete observation period except at point III.90(one-way ANOVA, *P*>0.05). We speculate that the change in alpha diversity on the 90^th^ day after drug withdrawal is a natural change, not the result of subcutaneous injection. In the CTR, CPZ, MEC and VAN groups, the observed OTU and Shannon index (Fig 1) clearly declined from the second day after drug delivery, which was significantly different compared to the control group (one-way ANOVA, *P*<0.05). These results are in accordance with the results of Knecht *et al*. [25], who observed that antibiotic therapy led to decreased alpha diversity that was not responsible for *C*. *difficile* colonization. In the OFL and AZI groups, the alpha diversity did not significantly differ from the control group. During the recovery period, we did not see an immediate return to normal microbiota for any antibiotic group. In the CTR, CPZ, MEC and VAN groups, the Shannon index and observed OTU returned to normal on the 14^th^ and 90^th^ days, respectively, after drug withdrawal, whereas the OFL and AZI groups returned to normal on the 4^th^day after drug withdrawal, suggesting a decreased dysbiosis effect. There has been extensive research carried out by many groups [26–32] on the dysbiosis effect of short-term and long-term antibiotic therapy, but fewer studies have focused on the recovery time of the gut microbiome after antibiotic therapy. We observed that the dysbiosis and recovery progress of alpha diversity in the CTR, CPZ, MEC and VAN groups were similar (one-way ANOVA, *P*>0.05), while OFL was similar with that of the AZI group. This may be because the former four antibiotics have a similar mode of action on the bacterial cell wall.

### Influence of antibiotics on beta diversity

Beta diversity means the dissimilarity between communities of two sites (or two samples). The higher beta diversity means the two communities are more dissimilar. In the CTR, CPZ, VAN and MEC groups, the intestinal microbiota changed significantly from the second day after drug delivery and returned to normal very slowly. The unweighted UniFrac of intestinal microbiota in the CTR, CPZ and MEC groups changed more obviously than in the control (CK) group because the antibiotics affected mostly the OTU with low abundance. UniFrac is used to determine whether communities are significantly different, to compare many communities simultaneously using clustering and ordination techniques. The weighted and unweighted UniFrac results (S1 and S2 Figs) were similar in both the OFL and AZI groups, which were highly similar to those of the CK group during the whole observation period. The weighted UniFrac result changed more obviously than the unweighted UniFrac result in the VAN group, which indicated that the VAN group mostly affected OTU with high abundance.

Then, we merged the data of each group at the same time point (S3 Fig), and we could see that CTR, VAN, CPZ and MEC have the strongest influence on intestinal microbiota, while OFL had the weakest influence, resembling the control group. It is worth noting that the intestinal microbiota of the AZI group did not change significantly during the drug delivery period and reached a change maximum after drug withdrawal. This indicates a delayed influence of AZI on the intestinal microbiota.

**Fig 2:**
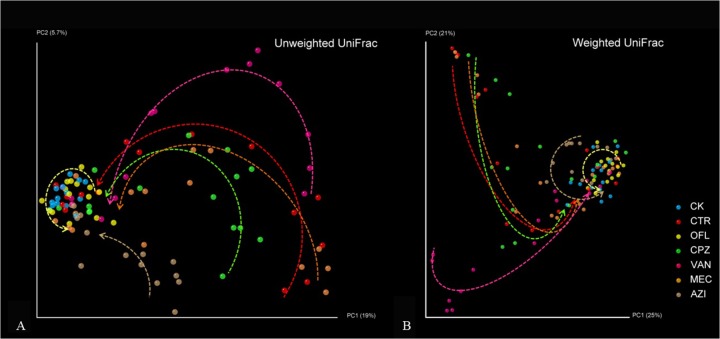
Principal coordinates analysis of unweighted and weighted UniFrac distances among different antibiotic groups. The starting points of the arrow lines are from the second day of antibiotic administration, and the arrow point is the end of observation period.

We additionally studied the PCoA of UniFrac distance among different antibiotic groups (Fig 2). We regard the position of drug delivery as the starting point and the end of the observation period as the terminal point. Each point represents a whole bacterial community, and the points far from each other represent lower similarity between the two communities. The weighted and unweighted UniFrac results of the OFL group did not indicate any changes, which is consistent with the UniFrac results that were affected by time. The influence of AZI was mild and was similar to the control group. The weighted UniFrac results clearly showed that the trajectories of the CTR, CPZ and MEC groups were similar, while the VAN group changed in accordance with the weighted UniFrac results of the VAN group (S2 Fig).

### Impact of different antibiotics on the gut microbial community structure

**Fig 3:**
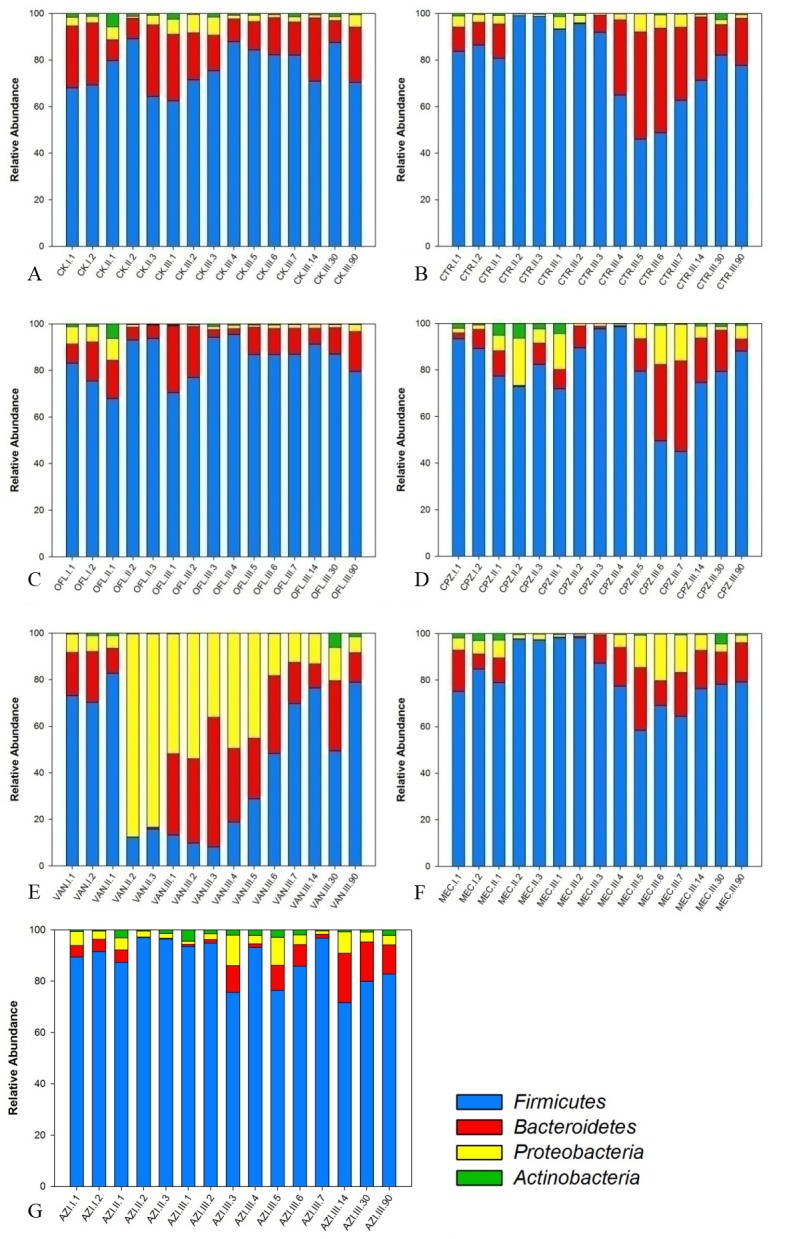
Microbial composition at the phylum level of distribution for different antibiotic groups. A.CK; B. CTR; C. OFL; D. CPZ; E. VAN; F. MEC; G AZI.I is sampling time before antibiotic treatment (I.1, I.2, I.3), II is the sampling during antibiotic administration (II.1, II.2, II.3, II.4), and III is after the antibiotic therapy (III.1, III.2, III.3, III.4, III.5, III.6, III.7, III.14, III.30, III.90). Here, 1, 2, 3, 4 and so on were sampling days.

After Illumina sequencing of the16srRNA and standardizing the sequences obtained, we detected 28 phyla. More than 98.2% of the sequences belonged to Firmicutes, Bacteroidetes, Proteobacteria and Actinobacteria (Fig 3). At the early stage of the drug delivery period, Firmicutes was the largest phyla among the control and antibiotic groups. The control group was stable during the whole observation period. β-lactam antibiotics interfere with cell wall synthesis by binding to penicillin-binding proteins located in bacterial cell walls, which leads to suppression of peptidoglycan synthesis and finally to cell death [33]. During the whole drug delivery period, we observed Firmicutes increased and Bacteroidetes and Proteobacteria decreased in CTR and MEC (β-lactam group), with a return to normal after drug withdrawal. However, in CPZ, Firmicutes decreased and Proteobacteria increased during the drug delivery period, and Firmicutes increased early and decreased later after drug withdrawal. Previous studies by Henrik *et al*. [25, 31] and Panda *et al*. [32] showed results similar to the CPZ group, whereas the CTR and MEC results were not in agreement with their results. Quinolones inhibit bacterial DNA gyrase and topoisomerase IV in gram-negative and gram-positive groups, respectively [34]. In the OFL group, Firmicutes, Bacteroidetes and Actinobacteria did not change throughout the observation period, whereas Proteobacteria decreased after drug delivery and remained low until the end of observation. Similar results were obtained by Panda *et al*. [32] after quinolone treatment in the human gut. In the VAN group, Firmicutes decreased from the second day after drug delivery, and Proteobacteria was the dominant phylum until drug withdrawal. During the recovery period, Bacteroidetes first rose and then returned to normal. A previous study by Anne *et al*. [35] showed similar results in vancomycin-treated gut microbiota. Vancomycin has a unique mode of action, including inhibiting the second stage of cell wall synthesis of susceptible bacteria, altering the permeability of the cell membrane and selectively inhibiting ribonucleic acid synthesis [36]. The change in the AZI group is not significant. Macrolides inhibit protein synthesis in the bacterial cell by preventing peptidyl transferase activity, as well as by inhibiting ribosomal translocation [37].

**Fig 4:**
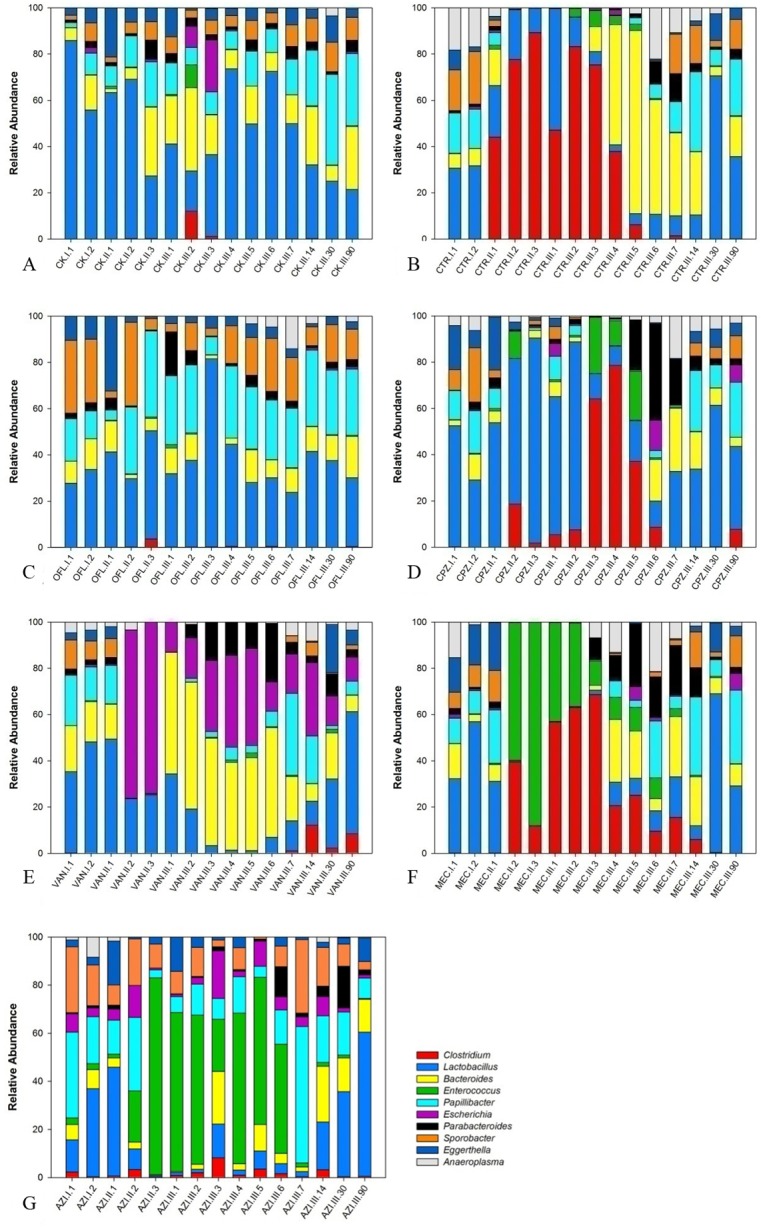
Microbial structure at the genus level for different antibiotic groups. A. CK; B. CTR; C. OFL; D. CPZ; E. VAN;F. MEC; G. AZI.

For the genus level comparison, we analyzed the top ten most abundant species. *Lactobacillus*, *Bacteroides*, *Papillibacter*, *Sporobacter*, *Eggerthella* were the dominant genera in most samples (Fig 4, S1 Table). Different antibiotic groups changed the gut microbiota in different ways. *Clostridium* rose soon after drug delivery in the CTR and MEC groups, while in the CPZ group, it reached a maximum level after drug withdrawal. *Papillibacter* and *Sporobacter* decreased in the CTR, MEC and CPZ groups. After drug withdrawal, *Clostridium* and *Lactobacillus* decreased and *Bacteroides* increased in the CTR group. In the CPZ group, *Lactobacillus* increased significantly and *Clostridium* rose and reached a maximum level after drug withdrawal, which was a delayed effect. There were no significant changes in the OFL group. In the VAN group, there was an increase in *Escherichia* from the second day after drug delivery until the end of the observation period. *Bacteroides* and *Parabacteroides* almost disappeared during VAN administration. *Lactobacillus* and *Eggerthella* decreased from the second day of drug delivery and continued decreasing until the 14^th^ day after drug withdrawal. This specific phenomenon was caused by VAN’s unique antibacterial spectrum and mechanism. Further studies are needed to determine whether VAN has a long-term impact on the intestinal microbiota in mice. *Bacteroides* (Bacteroidetes) and *Escherichia* (Proteobacteria) increased only in the VAN group during the drug delivery period, possibly because vancomycin eradicated most of the gram-positive bacilli.AZI had a slight impact on the intestinal microbiota, in which *Lactobacillus* and *Papillibacter* decreased after drug delivery while *Enterococcus* significantly increased until the second week after drug withdrawal. Increases in *Enterococcus*can lead to urinary tract infections, endocarditis and meningitis [38, 39] in humans.

**Fig 5:**
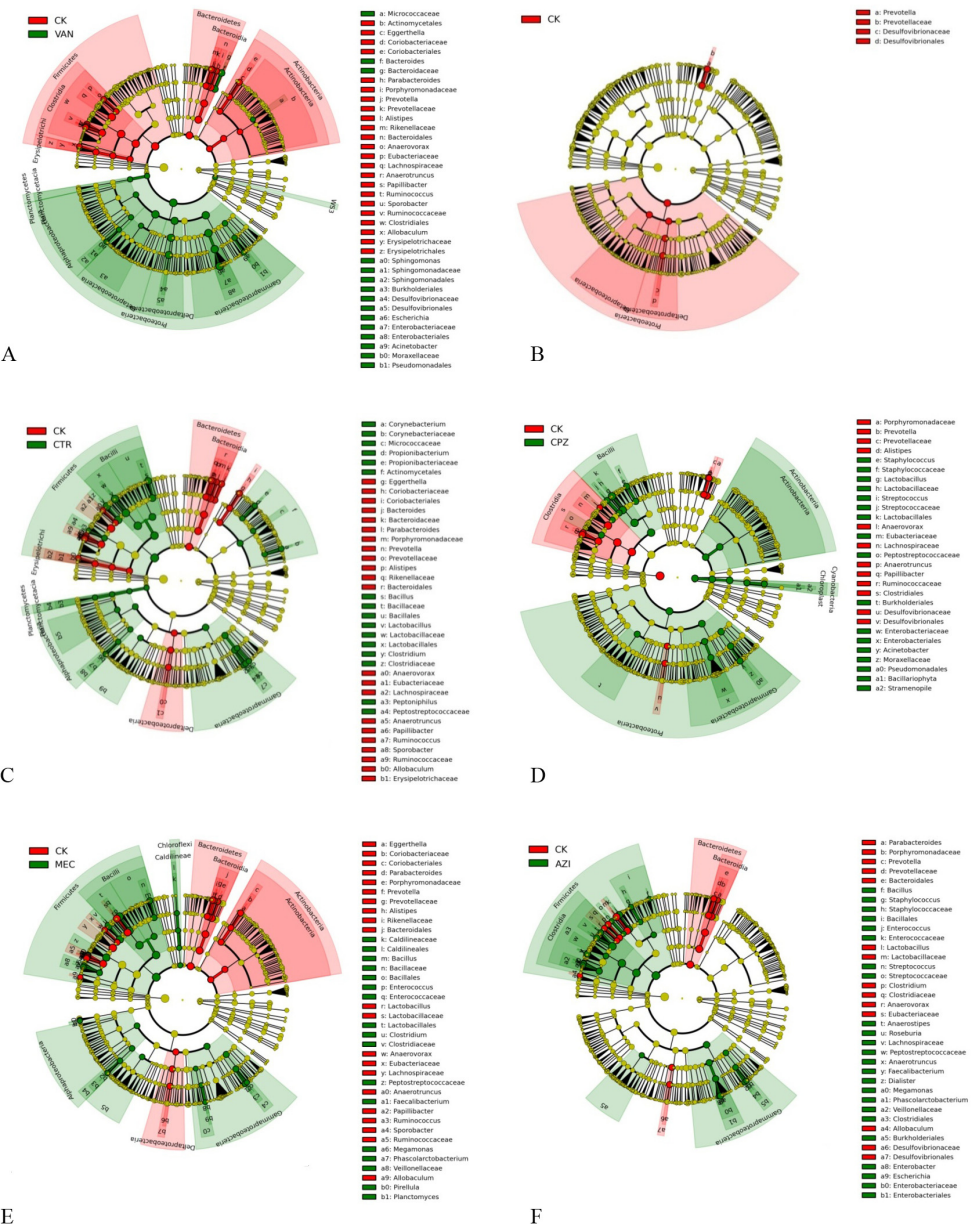
LEfSe comparison chart of control and antibiotic groups during antibiotic administration. A. VAN, B. OFL, C. CTR, D. CPZ, E. MEC and F. AZI.

**Fig 6:**
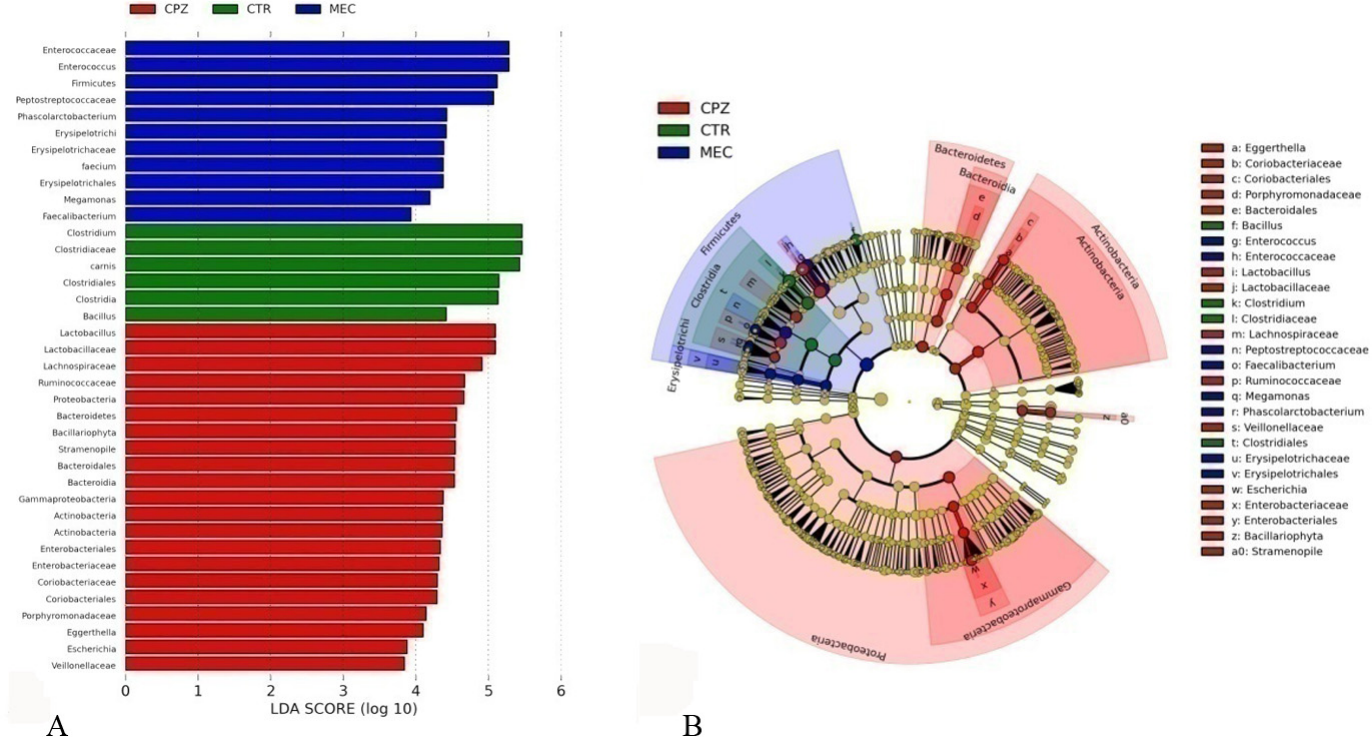
LEfSe statistic chart comparing β-lactams groups during antibiotic administration.

We used LEfSe to search the biomarkers of the metagenome using LDA analysis of nonparametric rank and inspection through the analysis of high-throughput sequencing data, providing biomarkers at different levels. We compared the changes in antibiotic groups with the control (CK) group during the drug delivery period (Fig 5). In all the antibiotic groups, there was a decline in *Bacteroidetes*, *Prevotella*, *Parabacteriodes* and *Porphyromonadaceae*. However, there was a proliferation of *Gammaproteobacteria*, mainly *Enterobacteriaceae*, *Pseudomonadales*, *Acinetobacter* and *Moraxellaceae*, except in the OFL group. The OFL group did not show any changes in gram-positive bacteria. Other than the VAN group, there were decreases in *Desulfovibrionaceae*, whereas *Escherichia* was increased only in the VAN group. Treatment with the three antibiotics belonging to the β-lactam category resulted in increased *Bacillus* and *Bacteroides*. These parallel shifts in the microbial community among different antibiotics were small. The OFL group had a very small effect on the mouse gut bacterial community structure. In this group, there was mainly decrease in *Beta* and *Deltaproteobacteria*. There were massive variations in bacterial communities among the different antibiotics. To further understand the effect of different antibiotics in the same category, we performed LEfSe (Fig 6) analyses for the β-lactam group. The bactericidal spectrum of CPZ is wider than CTR and MEC. In the CTR and MEC groups, Firmicutes was dominant, whereas in the CPZ group, Bacteroidetes, Actinobacteria and Proteobacteria were dominant. From this, we can conclude that antibiotics belonging to same category have distinct dysbiosis effects on the mouse gut microflora. It is well known that the gut microbiome of mouse model is different with that of human beings [40], which could be a limitation for extrapolating our results of the changes about specific genera or species. However, the general patterns, such as changes at high taxonomic levels and extend of alpha diversity, could be useful for future studies on the effect of antibiotics on gut microbiome of human and other animals.

### Conclusion

Changes in the gut microbial community structure and recovery time were distinct for different antibiotics, providing potential clues to aid in the clinical selection of antibiotics. To the best of our knowledge, this is the first comparative study focusing on the dysbiosis effect of different categories of antibiotics on the mouse gut microbiota and the associated recovery time. There is lot to explore in this field; we believe our findings will provide a baseline to carry out similar studies in humans.

## Supporting Information

S1 Fig PCoA of unweighted UniFrac distances among different antibiotic groups influenced by time factor. **A1.**CTR; **A2.**OFL; **A3.**CPZ; **A4.**VAN; **A5.**MEC;**A6.** AZI.
(TIF)

S2 Fig PCoA of weighted UniFrac distances among different antibiotic groups influenced by time factor. **B1.**CTR; **B2.**OFL; **B3.**CPZ; **B4.**VAN; **B5.**MEC;**B6.** AZI.
(TIF)

S3 Fig Principal coordinates analysis of unweighted (A) and weighted UniFrac (B) distances among different antibiotic groups influenced by time factor. (TIF)

S1 Table Significant differences in genus among antibiotic groups during antibiotic administration. S1 Table shows that *Bacteroides (Bacteroidetes)*and *Escherichia* (*Proteobacteria*) increased only in the VAN group during the drug delivery period. The reason may be that vancomycin killed most gram-positive bacilli. The influence of OFL was slight, and *Bacteroides* decreased only during the mid-drug administration period. AZI mainly eliminated *Bacteroidete*s, and it induced the enrichment of the OTUs with low abundance.
(DOCX)
