# Supplementary figures and images for: Different Dynamic Patterns of β-Lactams, Quinolones, Glycopeptides and Macrolides on Mouse Gut Microbial Diversity
Source: PLoS One. 2015 May 13;10(5):e0126712. doi: 10.1371/journal.pone.0126712 (PMC4430517; doi:10.1371/journal.pone.0126712)

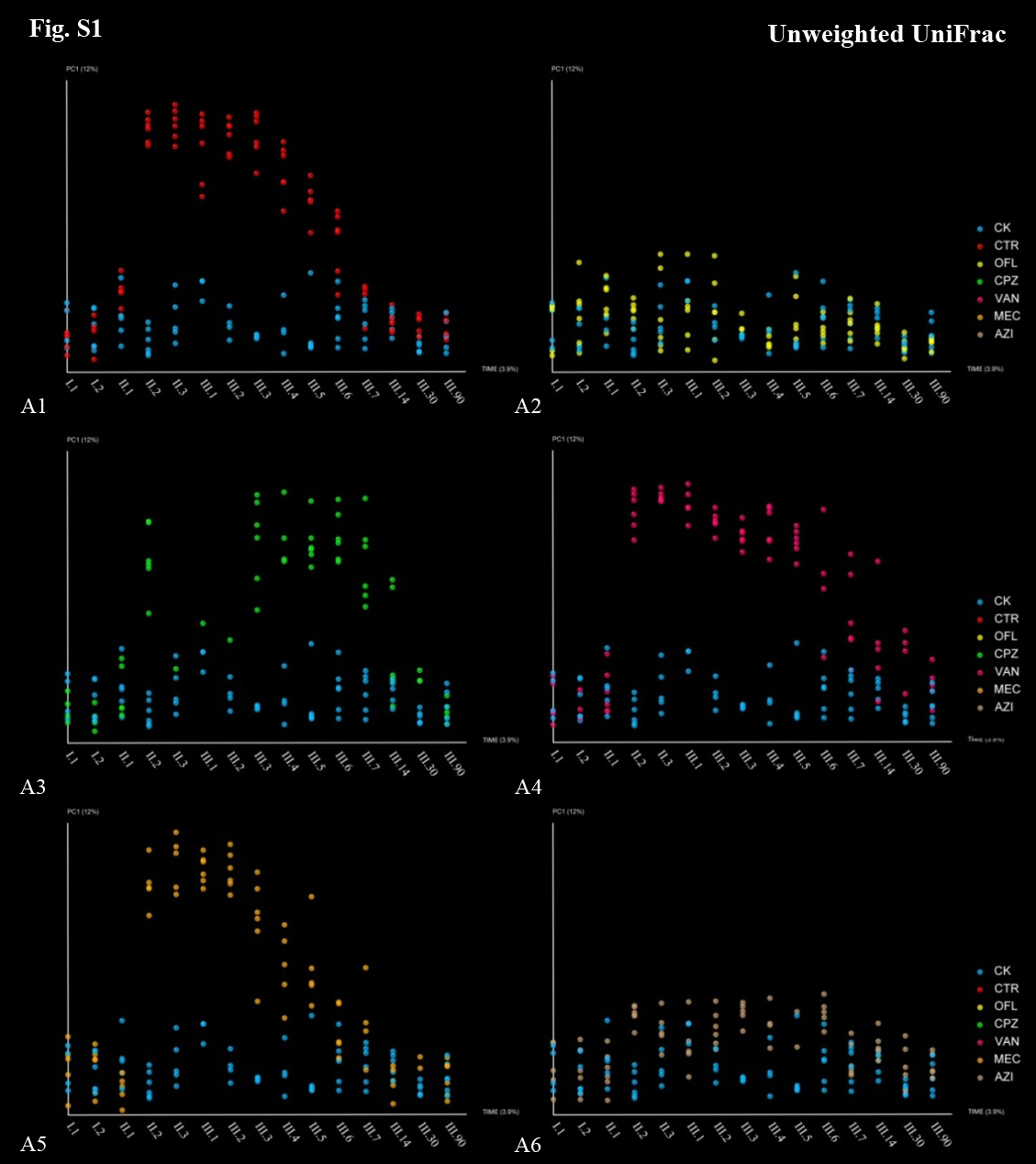

Supplement: S1 Fig — A1.CTR; A2.OFL; A3.CPZ; A4.VAN; A5.MEC;A6. AZI. (TIF) [file pone.0126712.s001.tif]

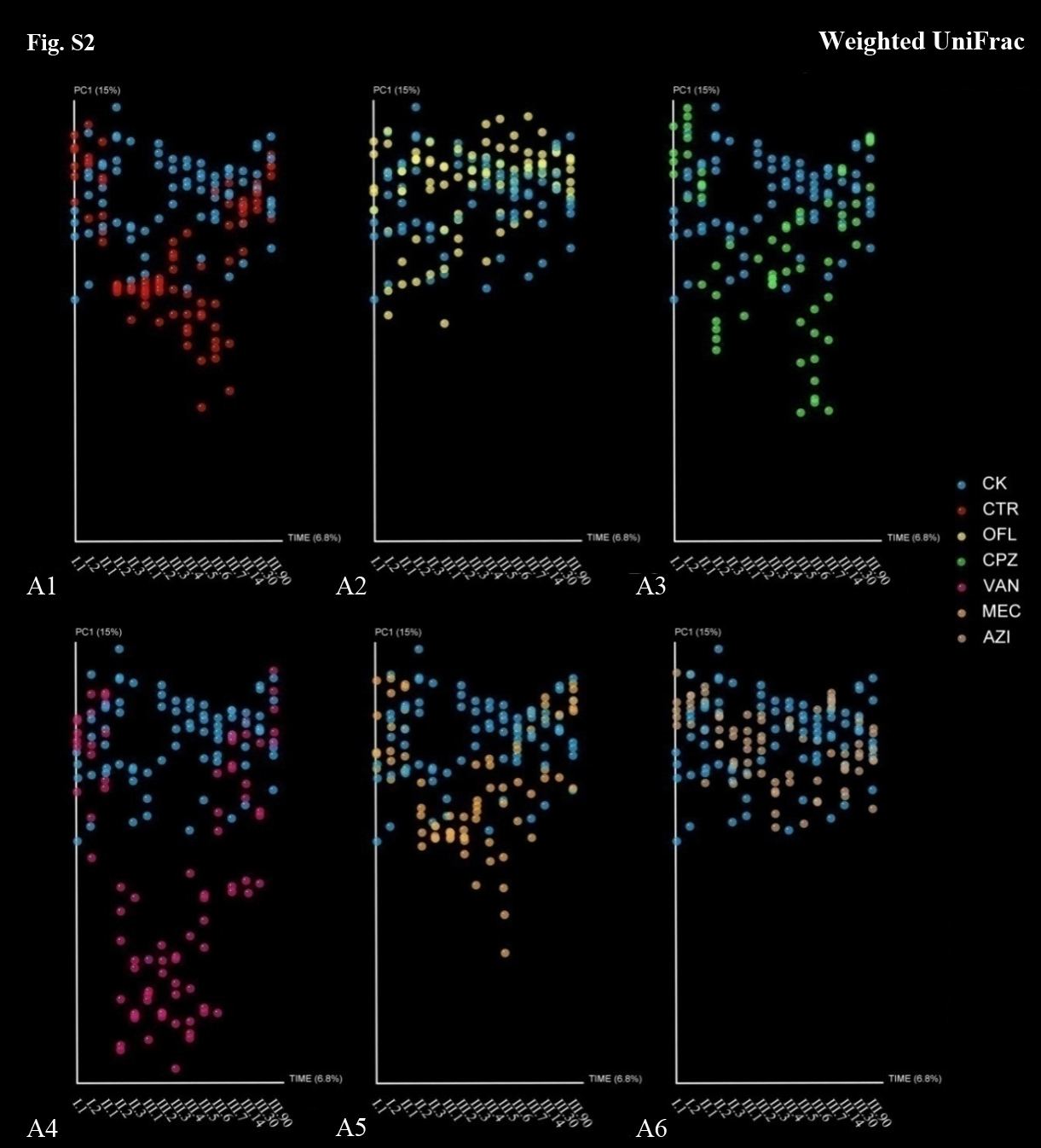

Supplement: S2 Fig — B1.CTR; B2.OFL; B3.CPZ; B4.VAN; B5.MEC;B6. AZI. (TIF) [file pone.0126712.s002.tif]

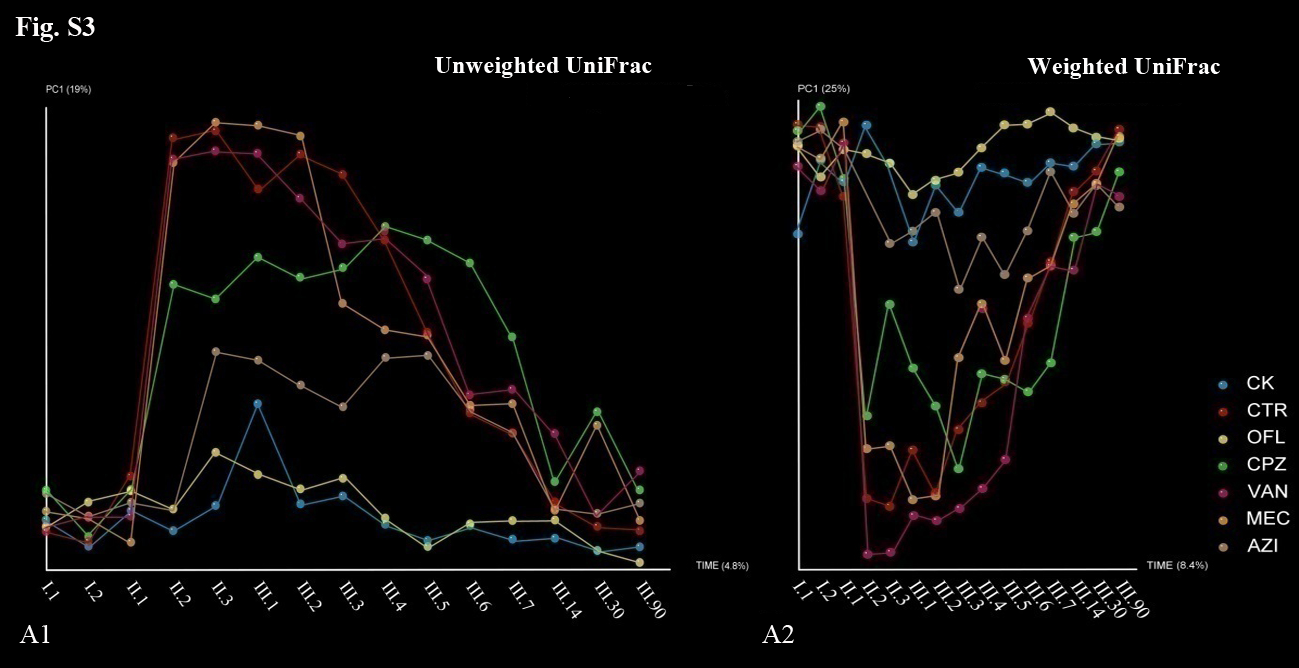

Supplement: S3 Fig — (TIF) [file pone.0126712.s003.tif]
