# Supplementary material for: Different Dynamic Patterns of β-Lactams, Quinolones, Glycopeptides and Macrolides on Mouse Gut Microbial Diversity
Source: PLoS One. 2015 May 13;10(5):e0126712. doi: 10.1371/journal.pone.0126712 (PMC4430517; doi:10.1371/journal.pone.0126712)
Supplement: S1 Table — shows that Bacteroides (Bacteroidetes)and Escherichia (Proteobacteria) increased only in the VAN group during the drug delivery period. The reason may be that vancomycin killed most gram-positive bacilli. The influence of OFL was slight, and Bacteroides decreased only during the mid-drug administration period. AZI mainly eliminated Bacteroidetes, and it induced the enrichment of the OTUs with low abundance. (DOCX) [file pone.0126712.s004.docx]

**Table S1**

| **AB** | **Increase** | **Decrease** |
| --- | --- | --- |
| CTR | ***F:*** *Lactobacillus*, *Clostridium, Bacillus, Peptoniphilus*  ***P:****Sphingomonas*, *Pseudomonas, AcinetobacterMoraxella*  ***A:****Corynebacterium, Propionibacterium* | ***F:*** *Papillibacter*, *Sporobacter, Ruminococcus,*  *Anaerotruncus, Allobaculum*  ***B:****Parabacteroides, Bacteroides, Prevotella,*  *Alistipes*  ***A:****Eggerthella* |
| CPZ | ***F:****Staphylococcus,Lactobacillus, Streptococcus*  ***P:****Acinetobacter* | ***F:****Papillibacter, Anaerovorax, Anaerotruncus*  ***B:****Prevotella, Alistipes* |
| MEC | ***F:****Clostridium,Bacillus, Enterococcus, Faecalibacterium,*  *Megamonas, Phasecolarctobacterium*  ***P:****Moraxella, Shewanella, Sphingomon*  *as, Acinetobacter* | ***F:****Lactobacillus*, *Sporobacter, Papillibacter,*  *Anaerovorax, Anaerotruncus,Allobaculum*,  *Ruminococcus*  ***B:****Parabacteroides*, *Alistipes, Prevotella*  ***A:****Eggerthella* |
| VAN | ***B:****Bacteroides*  ***P:****Sphingomonas, Escherichia, Acinetobacter* | ***F:****Papillibacter, Sporobacter,Ruminococcus,*  *Anaerovorax, Anaerotruncus*  ***B:****Alistipes, Prevotella, Parabacteroides*  ***A:****Coriobacteriaceae* |
| OFL |  | ***B:****Bacteroides*  ***P:****Delta proteobacteria, Beta proteobacteria* |
| AZI | ***F:****Bacillus, Staphylococcus,Enterococcus, Anaerostipes,*  *Roseburia, Faecalibacterium, Phascolarctobacterium,*  *Streptococcaceae,Anaerotruncus, Megamonas*  ***P:****Enterobacter, Escherichia, Moraxella, Acinetobacter* | ***B:****Parabacteroides, Prevotella* |
